# Analysis of the Risk Factors in Prognosis of Kawasaki Disease With Coronary Artery Lesions

**DOI:** 10.3389/fped.2021.798148

**Published:** 2021-12-07

**Authors:** Jinling Hu, Weidong Ren

**Affiliations:** Shengjing Hospital of China Medical University, Shenyang, China

**Keywords:** Kawasaki disease, coronary artery lesions, risk factors, prognosis, coronary artery disease

## Abstract

**Abstract:** Kawasaki disease (KD) is one of the most common forms of systemic vasculitis in children. Pathological features include extensive inflammation of small and medium blood vessels throughout the body. The primary complication of KD is coronary artery lesions (CALs). A total of 640 children with KD were admitted to the Department of Pediatric Cardiology at Shengjing Hospital of China Medical University from January 2017 to December 2019. These patients comprised 52 coronary artery aneurysm (CAA) cases and 47 coronary artery dilation (CAD) cases. Echocardiography was performed during the acute KD phase and then at 1, 3, 6, 12, and 24 months after KD onset. Patients were divided into a poor prognosis group (*n* = 30) and a normal group (*n* = 69) based on CALs prognosis. Differences in laboratory data, clinical manifestations and coronary artery damage rates were compared between the two groups. Univariate analysis was performed on these data, and an ROC curve was used to analyze the efficacy of each risk factor. Univariate analysis revealed that age (months), number of coronary arteries involved (NACI), IgM, IgA and brain natriuretic peptide (ProBNP) levels were higher in the poor prognosis group compared with the normal group, procalcitonin (PCT) levels in the poor prognosis group were lower than in the normal group (*P* < 0.05).

**Conclusion:** Age ≥ 18 months, IgM ≥ 1.07g/L, IgA ≥ 0.728g/L and NCAI ≥ 3 were poor prognostic factors of KD children with CALs. These parameters can be used as a reference indicator of early prediction where combined detection might improve the accuracy and sensitivity of prediction. Follow-up should be maintained to monitor changes in the coronary artery by echocardiography.

## Introduction

Kawasaki disease (KD) is one of the most common forms of systemic vasculitis in children (1). Pathological features include extensive inflammation of small and medium blood vessels throughout the body. The primary complication of KD is coronary artery lesions (CALs). When the coronary artery is involved, thrombosis, ruptured coronary aneurysm, myocardial infarction and heart failure may occur. These risk factors inevitable will affect the quality of life of children and even endanger their lives. Even after intravenous immunoglobulin (IVIG) treatment, CALs have been observed in 5–20% of patients with KD during the acute stage (2–4). KD complicated with CALs is the main cause of death. To our knowledge, this is the first study to explore the risk factors for prognosis of KD complicated with CALs. The purpose of this study was to elucidate a reliable method of predicting the prognosis in the early stage in order to provide a theoretical basis for the prognostic evaluation value of this disease.

### Patients

A total of 640 children with KD were admitted to the Department of Pediatric Cardiology at Shengjing Hospital of China Medical University from January 2017 to December 2019. Among them, 99 KD patients with complicating CALs were included. All children were diagnosed on the basis of the clinical criteria for KD (5). This study is a retrospective study, and it was approved and reviewed by the Ethics Committee of China Medical University.

The diagnostic criteria for complete KD (5) include a fever (>38°C) lasting at least 5 days without any other explanation, and at least four of the following criteria: polymorphous exanthema (rash of any kind), bilateral bulbar conjunctival injection without exudates, erythema of the oral mucosa (including lips, pharynx, or tongue), peripheral extremity changes (including erythema of palms or soles and/or swelling of hands or feet), and unilateral cervical lymphadenopathy (larger than 1.5 cm in diameter). Patients with only two or three principal clinical features of KD in addition to fever were considered to have incomplete KD when the other possible causes of fever have been excluded (6).

The M5S, X5, and S8 probes of GE VIVID E9 and Philips iE33 color Doppler ultrasonography were used, with probe frequencies of 1–5 and 2–8 MHz, respectively. Children in sleep or in a quiet state, take the supine or left decubitus position, routine position in the xiphoid process, apex, parasternal, suprasternal fossa to explore the standard section of the heart. Two pediatric ultrasonographers performed the echocardiographic examination of the left coronary artery (LCA), anterior descending artery (LAD), circumflex branches of the left coronary artery (LCX) and the main and distal segments of the right coronary artery (RCA) in order to determine whether there are any additional cardiac abnormalities. In this way, it is possible to exclude other diseases that can cause coronary artery dilatation and observe whether there are pericardial effusions, atrioventricular valve regurgitations, or any cardiac insufficiencies. The diameter and length of coronary arteries and coronary artery aneurysm (CAA) are measured at least three times and the average values are taken. In addition, it is important to discern whether there is a thrombosis in the tumor. Echocardiography is performed at acute phase, 1, 3, 6, 12, and 24 months after onset. The acute phase is defined as within 2 weeks of onset and no treatment is received.

CALs were assessed by echocardiography and were defined as coronary artery dilation (CAD): *Z-*score 2–2.5, small CAA: *Z-*score 2.5–5, medium CAA: *Z-*score 5–10 and internal diameter <8 mm, giant CAA (GCAA): *Z-*score ≥ 10, or internal diameter ≥8 mm (6).

Prognosis of CALs: The normal group: coronary artery returned to normal. The poor prognosis group: 1. no significant change; 2. worse than before.

### Clinical Examination

Data regarding epidemiology, clinical parameters and laboratory values were documented. Whole blood, plasma and serum samples were collected from all patients with acute KD before any treatment with intravenous immunoglobulin. These parameters included the following: age, gender, fever duration before IVIG, the number of involved coronary arteries (NCAI), oral change, conjunctival congestion, cervical lymphadenopathy, edema of the hands and feet, crissum flush desquamation, rush, erythema, IVIG resistance and KD classification (completely KD/incomplete KD), white blood cell count (WBC), hemoglobin (Hb), platelet count (Plt), neutrophil percentage (N%), eosinophils percentage (E%), C-reactive protein (CRP), erythrocyte sedimentation rate (ESR), alanine aminotransferase (ALT), aspartate aminotransferase (AST), albumin (ALB), lactate dehydrogenase (LDH), lymphocyte absolute value of absolute value of neutrophils ratio (NLR), platelet and the lymphocyte absolute value ratio (PLR), prothrombin time (PT), fribrinogen (FIB), fibrinogen degradation products (FDP), D-dimer, serum sodium, potassium, IgA, IgM and IgG, brain natriuretic peptide (N*T-*proBNP), procalcitonin (PCT) and IL-6.

### Statistical Analysis

All statistical analyses were performed using SPSS software version 23.0. Quantitative data were presented as the mean ± standard deviation, if the outcome measure distributions met the normality criteria. The independen*t-*sample Student's *t-*test was applied for comparing the means of continuous variables, while the abnormal distribution was represented by median (interquartile range) [M (p25–p75)], and the comparison between groups was conducted by the Mann–Whitney test. Qualitative data were expressed as percentages (*n*/%), and the chi-square test was used for comparison between groups. Receiver operating characteristic curves (ROC) were applied to determine the optimal cu*t-*off values of laboratory findings. Values were considered significantly different at *P* < 0.05.

## Results

**Figure 1:**
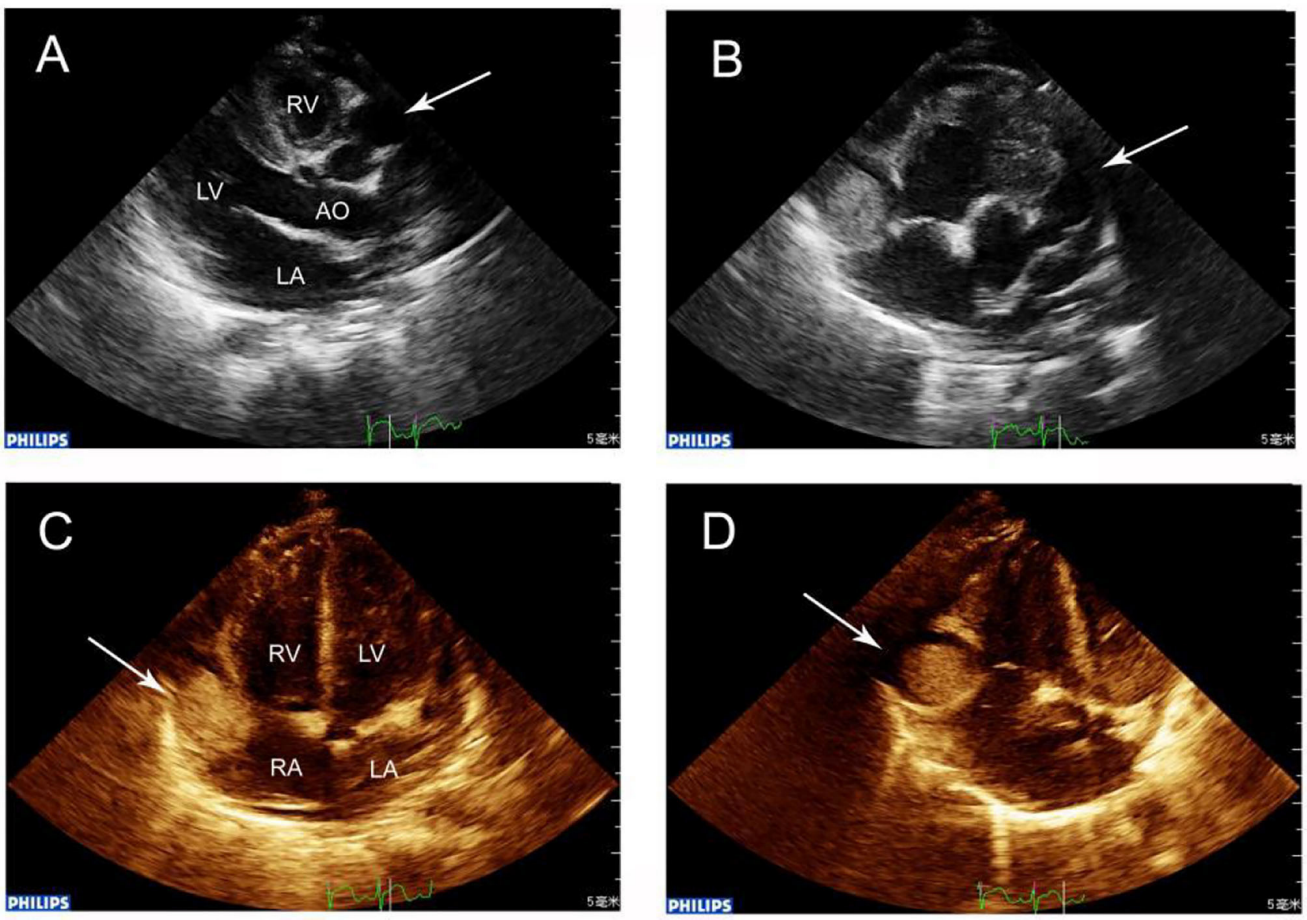
**(A)** Echocardiography showing RCA dilation (arrow) in the transthoracic long-axis view, showing the characteristic “string-of-beads” pattern. **(B)** Parasternal shor*t-*axis view showing a giant aneurysm (maximal diameter, 15.0 mm) in the RCA where within 5 mm, a thrombus was located. The LCA was also grossly dilated (arrow). **(C)** Apex four-chamber view revealing RCA that at the right atrioventricular ring had a giant aneurysm containing a thrombus (arrow). **(D)** In the giant aneurysm, a 13.0 × 13.0 mm thrombus was observed in echocardiography (arrow).

**Figure 2:**
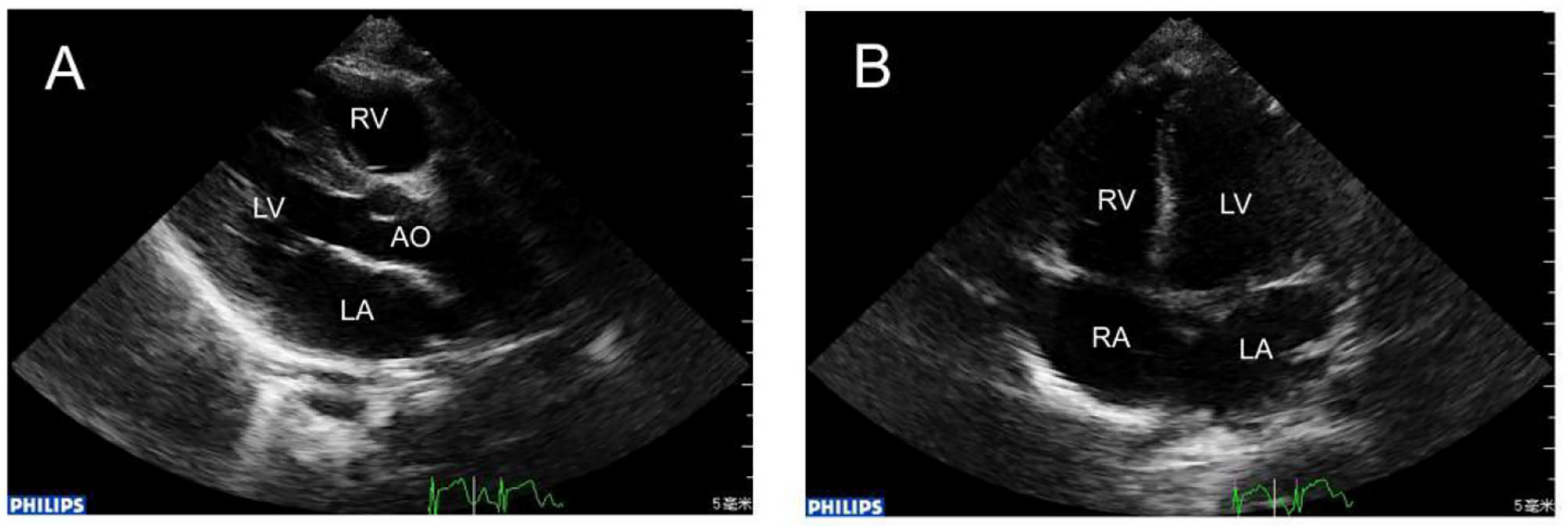
**(A)** 1 year later, repeat echocardiography showing that the RCA was in normal size. **(B)** The apex four-chamber view showed that in the same position the huge thrombus disappeared.

1. Results of echocardiography: Of the 99 KD patients with CALs, 52 patients were CAA and 47 patients were CAD. Echocardiography showed pericardial effusion in 13 patients, mitral regurgitation in five patients, tricuspid regurgitation in two patients, aortic valve regurgitation in one patient, two patients with large left heart, a reduced left ventricular posterior wall motion in one patient, and eight patients with thrombosis. After a 24-month follow-up, six patients showed that the thrombus still existed, one GCAA patient showed that the thrombus resolved, and the coronary artery returned to normal (Figures 1, 2). In one GCAA patient, the thrombus disappeared, but the coronary artery diameter did not recover. Coronary arteriography revealed coronary stenosis in two patients, and one of them developed myocardial infarction. No death was documented.

2. The time of occurrence and recovery of CALs: The initial incidence of CALs reached its peak within 1–2 weeks, 5.1% (5/99) developed CALs 1 month later. After 3 months, the initial incidence of CALs was very low. 95.7% (45/47) of CAD returned to normal within 1–3 months, and 28.8% (15/52) of CAA recovered within 3–6 months. The regression probability at 1, 3, 6, 12, and 24 months in the CAA group was 0, 15.4, 28.8, 38.5, and 42.3%, respectively, while in the CAD group, the regression probability was 46.8, 95.7, 100, 100, and 100%, respectively (Table 1). Of the 52 CAA patients, the NCAI was used to count the persistence rates. The data showed that at 12 months there were 18.5% (12/65 branches) for small CAA, 32.3% (21/65 branches) for medium CAA, 49.2% (32/65 branches) for GCAA, at 24 months there were 19.7% (12/61 branches) for small CAA, 31.1% (19/61 branches) for medium CAA, and 49.2% (30/61 branches) of GCAA (Table 2). The above studies showed that the persistence rate of small CAA in each period was better than the medium and the GCAA. Small CAA showed a faster recovery than the medium and the GCAA.

**Table 1:** The CALs status of all KD patients at different time points during the 2-year follow-up.

**Time after fever onset**	**CAA (*****n =*** **52)**		**CAD (*****n =*** **47)**	
	Persistent (%)^*^	**Regression (%)^#^**	Persistent (%)^*^	**Regression (%)^#^**
Acute phase	47 (47.5)	0 (0.0)	47 (47.5)	0 (0.0)
1 m	52 (52.5)	0 (0.0)	25 (25.3)	22 (46.8)
3 m	44 (44.4)	8 (15.4)	2 (2.0)	45 (95.7)
6 m	37 (37.4)	15 (28.8)	0 (0.0)	47 (100.0)
12 m	32 (32.3)	20 (38.5)	0 (0.0)	47 (100.0)
24 m	30 (30.3)	22 (42.3)	0 (0.0)	47 (100.0)

* *The percentage of lesions in the total sample*;

# *The percentage of recovered cases in the number of lesions*.

**Table 2:** CAA persistence rates according to the number of involved coronary arteries.

**Time after fever onset**	**CAA persistent**			**Total (*n*)**
	**Small, *n* (%)**	**Medium, *n* (%)**	**Giant, *n* (%)**	
Acute phase	20 (23.3)	33 (38.4)	33 (38.4)	86
1 m	27 (28.4)	34 (35.8)	34 (35.8)	95
3 m	19 (22.4)	33 (38.8)	33 (38.8)	85
6 m	15 (20.5)	26 (35.6)	32 (43.8)	73
12 m	12 (18.5)	21 (32.3)	32 (49.2)	65
24 m	12 (19.7)	19 (31.1)	30 (49.2)	61

3. The risk factors for prognosis: As shown in Table 3, univariate analysis revealed that age (*P* = 0.018), NCAI (*P* = 0.003), IgM (*P* = 0.019), IgA (*P* = 0.012), ProBNP (*P* = 0.006) and PCT (*P* = 0.006) levels were potential risk factors associated with CALs persistence.

**Table 3:** Univariate analysis for risk factors of prognosis.

	**The poor prognosis group (*n =* 30)**	**The normal group** **(*n =* 69)**	***P***
**Variables**			
Age (months)	29.5 (16.0–48.0)	17.0 (7.0–31.0)	0.018
**Sex**			0.091
Male (%)	25 (83.3%)	46 (66.7%)	
Female (%)	5 (16.7%)	23 (33.3%)	
**Classification**			0.055
Complete KD	23 (76.7%)	64 (92.8%)	
Incomplete KD	7 (23.3%)	5 (7.2%)	
Fever duration before IVIG	7.0 (5.0–13.0)	6.0 (5.0–10.0)	0.237
WBC	9.55 (6.78–12.64)	10.70 (8.34–13.69)	0.208
HB	103.65 ± 15.55	101.90 ± 14.50	0.590
N	3.75 (1.65-8.25)	5.50 (3.05–9.40)	0.209
PLT	285.50 (224.50–496.00)	417.00 (279.00–641.00)	0.328
L	3.15 (2.50–4.67)	3.50 (2.20–5.20)	0.645
NLR	1.10 (0.44–1.97)	1.56 (0.74–3.00)	0.112
PLR	111.18 (65.96–173.04)	112.73 (71.04–168.91)	0.565
N%	45.82 ± 23.71	51.21 ± 21.58	0.271
E%	2.40 (0.75–3.88)	2.10 (0.80–5.85)	0.799
CRP	37.45 (16.55–81.35)	73.70 (21.55–103.00)	0.105
ESR	56.50 (25.00–87.80)	60.00 (37.50–81.00)	0.233
AST	23.50 (18.00–33.00)	25.00 (18.50–31.00)	0.763
ALT	22.50 (10.00–39.50)	23.00 (14.50–49.00)	0.178
LDH	271.00 (228.75–325.25)	274.00 (226.00–351.00)	0.784
GGT	32.00 (13.75–106.75)	48.00 (19.50–85.50)	0.602
TB	4.70 (3.40–7.23)	4.70 (3.25–6.50)	0.324
TBA	5.80 (3.80–9.68)	6.30 (3.70–12.60)	0.576
ALB	31.98 ± 4.91	31.59 ± 5.21	0.724
A/G	0.95 (0.60–1.50)	0.80 (0.65–1.10)	0.360
Na	136.00 (134.75–138.00)	136.00 (133.50–137.50)	0.435
K	4.47 ± 0.54	4.35 ± 0.72	0.420
IgG	16.22 ± 9.85	17.32 ± 11.28	0.645
IgM	1.29 (0.77–1.72)	0.97 (0.63–1.30)	0.019
IgA	1.00 (0.70–1.45)	0.58 (0.42–1.05)	0.012
PT	13.55 (12.38–14.90)	12.90 (12.30–14.00)	0.136
FIB	4.05 (2.45–5.25)	4.20 (3.55–4.96)	0.241
FDP	5.15 (2.08–10.35)	5.80 (3.10–10.15)	0.402
D-dimer	347.50 (239.50–1,033.25)	598.00 (329.00–1,105.00)	0.099
PCT	0.16 (0.11–0.58)	0.54 (0.18–1.42)	0.006
proBNP	593.15 (150.13–1,700.25)	501.40 (245.85–1,935.50)	0.006
IL6	38.85 (26.41–191.90)	88.53 (37.06–196.55)	0.743
VD	37.26 ± 12.71	34.39 ± 12.06	0.288
Conjunctival congestion	21 (70.0%)	52 (75.4%)	0.577
Oral change	20 (66.7%)	58 (84.1%)	0.052
Rush	15 (15.0%)	46 (66.7%)	0.117
Edema of the hands and feet	16 (53.3%)	35 (50.7%)	0.811
Cervical lymphadenopathy	21 (70.0%)	48 (69.6%)	0.965
Crissum flush desquamation	9 (30.0%)	18 (26.1%)	0.688
IVIG resistance	22 (73.3%)	55 (79.7%)	0.483
**NCAI**			0.003
1	7 (23.3%)	32 (46.4%)	
2	10 (33.3%)	29 (42.0%)	
3	12 (40.0%)	8 (11.6%)	
4	1 (3.3%)	0 (0.0%)	

**Figure 3:**
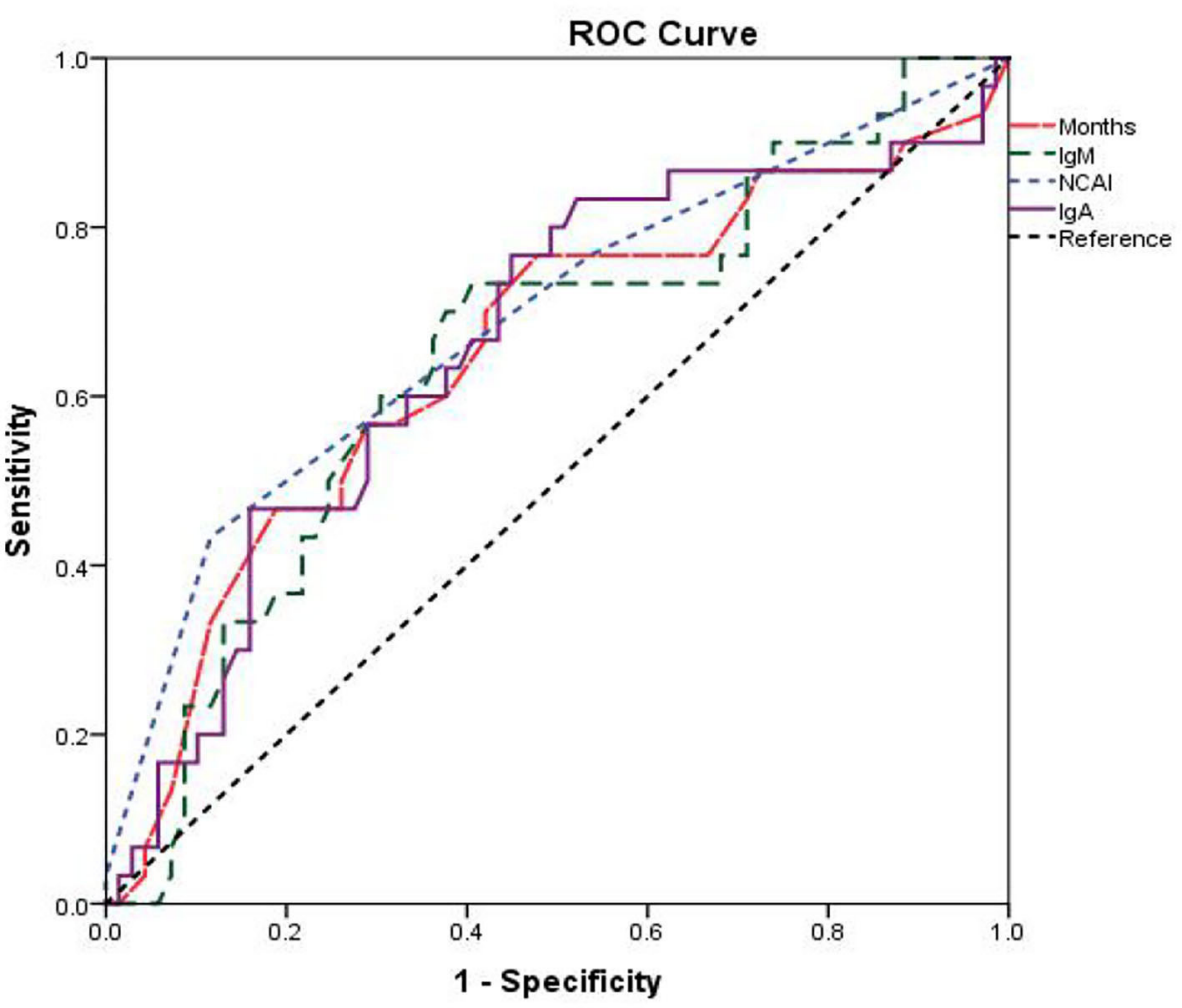
The ROC curve of age, IgM, IgA, and NCAI independently predicts CALs.

The area under curve (AUC) and cu*t-*off values were calculated (Table 4). The results showed that the cu*t-*off value of age for KD diagnosis was 18 months, AUC was 0.65, sensitivity and specificity were 76.67 and 52.18%, respectively, *P* < 0.05. The cu*t-*off value of NCAI was 3, AUC was 0.689, sensitivity and specificity were 43.33 and 88.41%, respectively, *P* < 0.05. The cu*t-*off value of IgM was 1.07 g/L, AUC was 0.648, sensitivity and specificity were 73.33 and 59.42%, respectively, *P* < 0.05. The cu*t-*off value of IgA was 0.728 g/L, AUC was 0.659, sensitivity and specificity were 76.67% and 55.07%, respectively, *P* < 0.05. The cu*t-*off value of proBNP was 7,360 ng/L, AUC was 0.521, sensitivity and specificity were 13.33 and 97.1%, respectively, *P* > 0.05. The cu*t-*off value of PCT was 0.063 ng/mL, AUC was 0.676, the sensitivity and specificity were 100 and 4.3%, respectively, *P* < 0.05. The ROC curves were drawn by age, NACI, IgM, IgA, ProBNP, and PCT (Figure 3).

**Table 4:** Single variable predicts the evaluation of CALs.

**Variables**	**Cu*t-*off**	**AUC (95%CI)**	***P***	**sensitivity**	**specificity**
Age (months)	≥18	0.650 (0.526–0.774)	0.018	76.67%	52.18%
IgM	≥1.07	0.648 (0.529–0.767)	0.019	73.33%	59.42%
IgA	≥0.728	0.659 (0.538–0.780)	0.012	76.67%	55.07%
PCT	≤ 0.0630	0.676 (0.554–0.797)	0.006	100.00%	4.30%
proBNP	≤ 7360	0.521 (0.391–0.651)	0.743	13.33%	97.10%
NCAI	≥3	0.689 (0.570–0.808)	0.003	43.33%	88.41%

**Figure 4:**
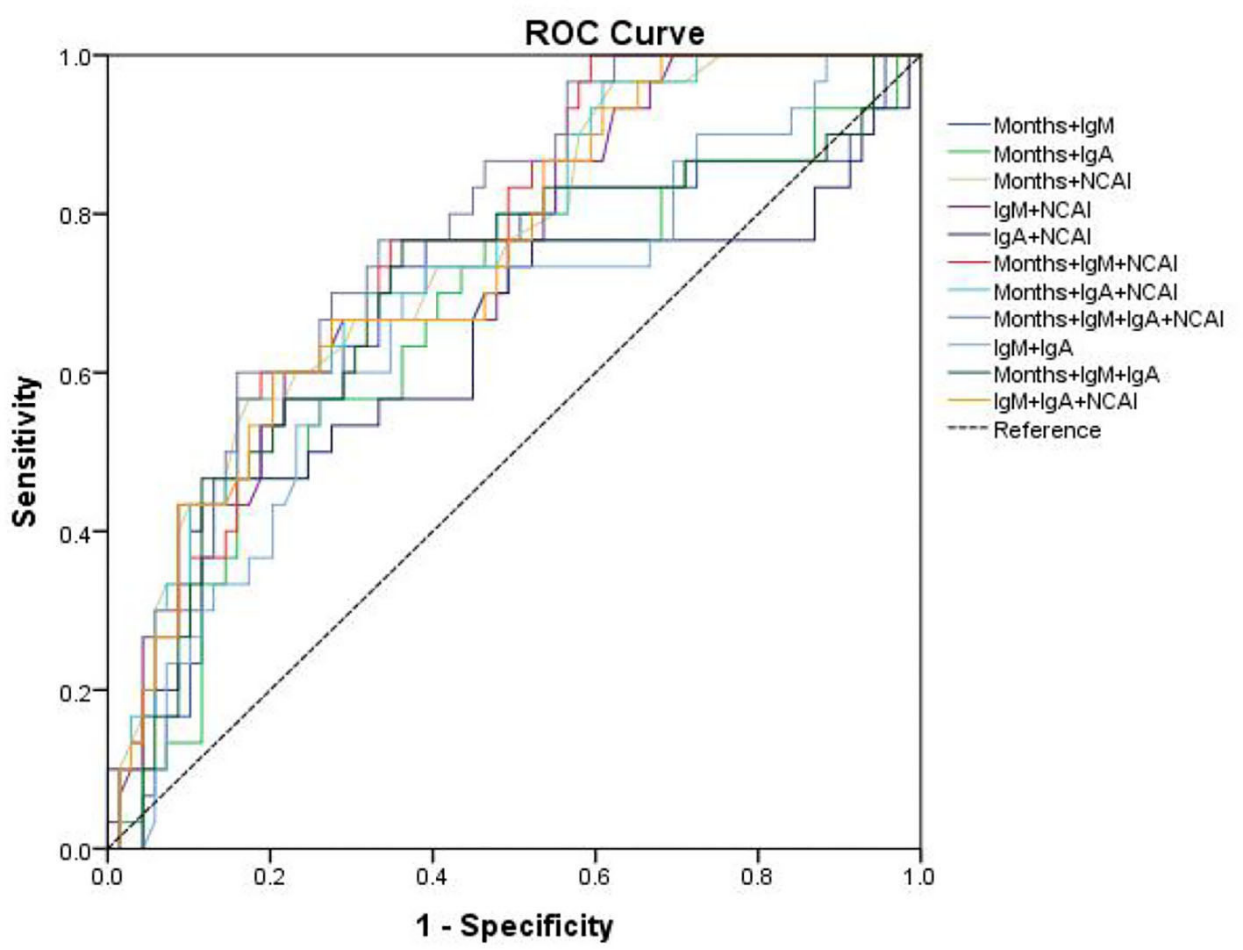
The ROC curve of age, IgM, IgA and NCAI combined prediction of CALs.

The sensitivity and specificity of combined prediction among different indicators are shown in Table 5. The best AUC value of age, NACI, IgM and IgA was 0.777, yielding a sensitivity and specificity of 60.0 and 84.06%, respectively. The ROC curve is shown in Figure 4.

**Table 5:** Combined diagnosis of the risk factors.

**Variables**	**AUC (95%CI)**	***P***	**sensitivity**	**specificity**
age+IgM	0.684 (0.562–0.805)	0.004^*^	73.33%	65.22%
age+IgA	0.653 (0.530–0.775)	0.016^*^	46.67%	84.06%
age+NCAI	0.750 (0.648–0.851)	<0.001^*^	56.67%	82.61%
IgM+NCAI	0.735 (0.632–0.839)	<0.001^*^	60.00%	78.26%
IgA+NCAI	0.628 (0.495–0.762)	0.068	46.67%	86.96%
IgM+IgA	0.662 (0.544–0.780)	0.011^*^	73.33%	60.87%
age+IgM+NCAI	0.767 (0.672–0.861)	0.048^*^	76.67%	65.22%
age+IgA+NCAI	0.754 (0.654–0.853)	<0.001^*^	56.67%	84.06%
age+IgM+IgA	0.695 (0.575–0.815)	0.002^*^	76.67%	63.77%
IgM+IgA+NCAI	0.743 (0.641–0.845)	0.052	60.00%	79.71%
age+IgA+IgM+NCAI	0.777 (0.685–0.870)	0.047^*^	60.00%	84.06%

* *P < 0.05*.

## Discussion

KD is an acute self-limited vasculitis, principally affecting coronary arteries in children under 5 years old (7). The etiology of the disease is still unknown, even though many studies continue to investigate the possible triggers of this vasculitis. Many studies have confirmed that in children with KD, more than 20% of patients with CALs and ~10% of children will have myocardial infarction, sudden death due to coronary artery stenosis and thrombosis. CALs are the most serious complication of KD. They can last for many years and are a potential risk factor for coronary heart disease in adulthood.

Echocardiography can serve as an early detection of CALs. Follow-up observation of the disease outcome, especially in children presenting with a thinner chest wall, is useful for better monitoring of the proximal coronary artery. In this study, the incidence of CALs reached its peak within 1–2 weeks, where 5.1% developed CALs 1 month later. Therefore, follow-up should not be given in acute phase patients without coronary artery disease. However, the risk of recurrence of coronary artery disease is very low in children with a normal coronary artery as detected by echocardiography 3 months after the onset of the disease. Clinicians can adjust the time of treatment and plan accordingly. Advani et al. (7) found that 77.4% of small and medium-sized CAAs could return to normal within 2 years of the onset of the disease, while GCAAs tended to expand continuously. In the present study, during a 2-year follow-up, the persistent rates of CAA were 19.7% in the small CAA group, 31.1% in the medium CAA group and 49.2% in the GCAA group. It is suggested that the prognosis of small CAA is better than the medium and the GCAA. However, even if the diameter of the CAA retracts to normal, its intimal proliferation and vascular regeneration can still last for several years. Coronary artery stenosis, coronary artery diastolic dysfunction, and intimal thickening may still lead to coronary atherosclerosis and myocardial ischemia in adolescents (8). Some children can still present with an enlarged CAA, coronary artery calcification, recanalization, and segmental stenosis after thrombus occlusion in the adult stage (9). In this study, eight cases of thrombosis and one case of myocardial infarction were found, all of which occurred within 2 years of the disease course. Myocardial infarction leads to the deterioration of left ventricular function and is thus the worst prognosis. The greater the peak diameter of CAA, the higher the GCAA number, and the more coronary arteries involved in thrombosis, the more difficult it is for patients to recover from coronary artery damage, and the worse the prognosis. For such children, early treatment and long-term close follow-up are recommended to monitor any occurrences of cardiovascular events.

In addition to observation that the diameter and the prognosis of CALs are closely related, it was found that the average age of the normal group was 17 months, whereas the poor prognosis group was 29.5 months. The age difference between the two groups was statistically significant. The ROC curve suggested that patients older than 18 months might have a poor prognosis. Takahashi et al. (10) suggested that the difference in age might be related to the stronger function of vascular reconstruction and repair at a younger age. However, the specific mechanism still needs further study.

A previous study (11) showed that abnormal activation of T cells was the initiation and key step of vascular immune injury. The abnormal activation of T cells and monocytes in the peripheral blood can release inflammatory mediators and cytokines, thus promoting the transformation of B cells into plasma cells. This results in an increase of serum IgG, IgM, IgA, and IgE that might damage the vascular endothelial barrier through complement dependent cytotoxicity. Similarly, intravenous injection of IVIG may inhibit the activation of innate immune cells such as dendritic cells and neutrophils, while also inhibiting the secretion of inflammatory mediators. This combined immunomodulatory effect could enhance the regulation of T lymphocytes (Treg), and achieve the therapeutic effect of KD (12). Rowley et al. (13) reported the vascular IgA response in acute KD was oligoclonal. The identification of an oligoclonal IgA response in KD strongly suggested that the immune response to this important childhood illness was Ag-driven. Kim et al. (14) studied the effect of immunoglobulin isotype on inflammatory data and clinical outcomes in 241 patients with KD, and found that high IgA levels were prognostic for the risk of CALs. In our study, there was a significant increase in IgM and IgA in the poor prognosis group, which also confirmed that the occurrence of KD might related to an immune disorder.

PCT is a recently discovered sepsis marker discovered that is significantly increased in severe bacterial infection and inflammation. PCT levels begin to increase 2–3 h after infection, and reaches expression peak at 24–48 h. The specificity of PCT is higher than CRP and ESR. Previous studies (15, 16) have shown a significant difference in PCT level between the coronary artery disease group and the non-coronary artery disease group, where the former showed an increase in the PCT level. However, Samuel et al. (15) suggested that PCT could not be used as a predictor of coronary artery injury. Taken together, the above studies indicate that the significance of PCT levels in KD is inconclusive. In the present study, PCT in the poor prognosis group was lower than in the normal group. The reason might be due to the fact that the KD children in this study were complicated with bacterial and/or mycoplasma infection during hospitalization (the infection rate was 90.9%, 90/99). In this case, the PCT levels might have been related to infection. Thus, due to of the inconclusive nature of the results, PCT was not included in the combined diagnosis index.

## Limitations

This study has several limitations. A limited number of patients were enrolled and selection bias may have affected the accuracy of the statistical results. And the difference of size of CALs between the two groups might affect the results.

## Conclusions

Even if the diameter of blood vessels return to normal, changes in vascular pathology and function increase the risk of cardiovascular events in children. Therefore, once CALs form, long-term or even life-long follow-up should be conducted, in particular for GCAA patients. At present, there are very few studies on predicting the risk factors of prognosis in KD children with CALs. The present study showed that there are certain limitations in predicting the prognosis of age, IgM, IgA, and NCAI alone. The AUC was largest when the four factors were combined, and the predictive value of prognosis was the highest among any other combination of indicators.

## Data Availability Statement

The original contributions presented in the study are included in the article/supplementary material, further inquiries can be directed to the corresponding author/s.

## Ethics Statement

This study is a retrospective study, and it was approved and reviewed by the Ethics Committee of China Medical University. The ethics code is 2021PS537K. Informed consent was obtained from all subjects or, if subjects are under 16, from a parent and/or legal guardian. And all methods were performed in accordance with the relevant guidelines and regulations. Written informed consent to participate in this study was provided by the participants' legal guardian/next of kin. Written informed consent was obtained from the minor(s)' legal guardian/next of kin for the publication of any potentially identifiable images or data included in this article.

## Author Contributions

JH wrote the manuscript and prepared the figure. All authors reviewed the manuscript.

## Conflict of Interest

The authors declare that the research was conducted in the absence of any commercial or financial relationships that could be construed as a potential conflict of interest.

## Publisher's Note

All claims expressed in this article are solely those of the authors and do not necessarily represent those of their affiliated organizations, or those of the publisher, the editors and the reviewers. Any product that may be evaluated in this article, or claim that may be made by its manufacturer, is not guaranteed or endorsed by the publisher.
